# Exploring the relationship between teacher growth mindset, grit, mindfulness, and EFL teachers’ well-being

**DOI:** 10.3389/fpsyg.2023.1241335

**Published:** 2023-09-25

**Authors:** Jianyu He, Shisir Iskhar, Yan Yang, Moldobaeva Aisuluu

**Affiliations:** ^1^School of Foreign Languages, Luoyang Normal University, Luoyang, Henan, China; ^2^Kyrgyz-Chinese Department, Kyrgyzstan State University, Bishkek, Kyrgyzstan; ^3^National Academy of Sciences of the Kyrgyz Republic, Bishkek, Kyrgyzstan

**Keywords:** teacher growth mindset, mindfulness, grit, occupational well-being, mediation, EFL teachers, structural equation modeling

## Abstract

**Introduction:**

This study examines the relationship between teacher growth mindset, mindfulness, grit, and teacher well-being, with a particular emphasis on the mediating role of grit.

**Methods:**

The study involved 547 Chinese EFL teachers as participants. Data collection utilized validated measures of growth mindset, mindfulness, grit, and occupational well-being. Structural equation modeling was employed to analyze the data and investigate the proposed relationships.

**Results:**

The findings reveal several important relationships. Firstly, both teacher growth mindset and teacher grit exhibit a direct positive influence on teacher well-being. Secondly, teacher grit acts as a mediator in the connection between teacher mindfulness and teacher occupational well-being. This suggests that the positive impact of mindfulness on well-being is, in part, explained by the presence of grit.

**Discussion:**

These findings significantly contribute to our comprehension of the factors influencing teacher well-being. They underscore the importance of cultivating growth mindset, mindfulness, and grit in educational contexts. Moreover, the implications of these findings for teacher training and support programs are discussed.

## Introduction

Educators wield a profound influence over learners’ academic, societal, and emotional achievements (King and Chetty, 2014). The link between learners’ engagement, motivation, academic performance, and teaching practices is well-established (Bartholomew et al., 2018; Granziera et al., 2023). As a result of the growing research interest in the subject of occupational well-being over the past few years, teacher well-being in the context of their profession has been acknowledged as a crucial determinant for the well-being of learners, educational accomplishments, and teachers’ dedication to their profession (Chang and Cherng, 2017; Harding et al., 2019). Teacher well-being, encompassing professional fulfillment and satisfaction, is multifaceted, embracing both general and domain-specific dimensions (Acton and Glasgow, 2015; Diener et al., 2017). Psychological perspectives, including mindset, grit, and mindfulness, shape individuals’ perceptions, attitudes, and overall well-being, influencing their professional engagement and academic success (Dweck, 2006; Nalipay et al., 2021). A growth mindset, as proposed by Dweck (2006), pertains to one’s belief in the malleability of their abilities. Teachers with growth mindsets tend to be more engaged and experience higher well-being (Zeng et al., 2019; Liu et al., 2023). In parallel, teacher grit, defined as unwavering dedication to teaching despite challenges, contributes to feelings of fulfillment and student success (Maiers and Sandvold, 2017; Derakhshan et al., 2022). Grit aligns with a growth mindset (Suzuki et al., 2015), reinforcing the notion that EFL instructors’ grit can enhance their engagement and satisfaction in teaching (Duckworth et al., 2009; Hill et al., 2016; Fathi et al., 2023).

Mindfulness, another focal variable, plays a pivotal role in teacher well-being (Hassed and Chambers, 2014; Frank et al., 2016; Li, 2021). Rooted in Buddhist philosophy, mindfulness emphasizes conscious awareness of both internal and external experiences (Brown and Ryan, 2003). In the demanding realm of second language education, where instructors grapple with contextual, technical, psychological, and affective pressures (Li, 2021), mindfulness becomes especially relevant. Numerous educational factors, including well-being, academic performance, engagement, and teaching effectiveness, are linked to mindfulness (Kee and Liu, 2011; Hue and Lau, 2015; Rosenstreich and Margalit, 2015; Bartlett et al., 2019). Mindfulness practices alleviate fatigue, anxiety, and depression among instructors (Harnett et al., 2016; Li, 2021).

Although individual studies have examined the impact of teacher growth mindset, mindfulness, and grit on teacher occupational well-being separately, there is a lack of research that integrates these constructs within a comprehensive model. Understanding the interrelationships between these factors can provide a more nuanced understanding of teacher occupational well-being and identify potential points of intervention to enhance teacher well-being. As a result, the present study aims to investigate the relationships between teacher growth mindset, mindfulness, grit, and teacher occupational well-being among Chinese EFL teachers. Specifically, we hypothesize that teacher growth mindset positively influences teacher occupational well-being, that teacher mindfulness positively influences teacher occupational well-being, and that teacher grit mediates the relationship between mindfulness and well-being. Additionally, we explore the direct effects of teacher growth mindset and grit on teacher well-being. By examining these relationships, this study contributes to the existing literature on teacher occupational well-being and provides insights into the factors that can promote positive well-being among EFL teachers.

## Literature review

### Positive psychology

Positive psychology (PP), introduced by Seligman (2006), offers an alternative to student-centered education, rooted in philosophical psychology. It delves into how emotions and sentiments can facilitate personal growth, ultimately enhancing individuals’ quality of life (MacIntyre et al., 2019; Li, 2021). PP emerged partly as a response to the dominance of cognitivist attitudes in second-language acquisition (SLA), which often downplayed the role of emotions in L2 learning (Seligman, 2006; Mongrain and Anselmo-Matthews, 2012). This approach encourages individuals to embrace life’s positive aspects rather than dwelling solely on its challenges and setbacks, without disregarding obstacles and failures (Oxford, 2016). PP centers on human strengths and capabilities rather than flaws. Seligman (2011) identifies its core components as positive feelings, positive personality traits associated with well-being, and supportive environments that foster growth. In the context of second language acquisition, PP has gained prominence, with studies demonstrating its capacity to enhance EFL instructors’ and learners’ efficacy, resilience, engagement, motivation, creativity, and mitigate the impact of negative emotions (Mercer, 2017; Dewaele et al., 2019; MacIntyre et al., 2019; Fathi et al., 2023; Zhang et al., 2023).

### Well-being

According to Diener et al. (2017), teacher well-being is seen as having several facets and dimensions and includes the professional fulfillment and satisfaction of teachers (Acton and Glasgow, 2015; Li et al., 2022). Research on teachers’ well-being typically focuses on their work satisfaction or on their general well-being, particularly how personal characteristics and environmental elements at the school are related to teachers’ occupational well-being (Diener, 2000; Collie and Martin, 2017; Vorkapić and Peloza, 2017; Greenier et al., 2021; Xiyun et al., 2022). Nevertheless, according to the broaden-and-build theory (Fredrickson and Levenson, 1998) and the understanding from earlier teacher wellbeing models, immediate emotional experiences—both positive and negative—are first assessed by past behavior, which then either reinforces or depowers individual capacities (Spilt et al., 2011; Frenzel, 2014; Li et al., 2022). Momentary emotional events then affect a person’s general perception of life by improving or hindering their wellbeing in a particular category. Positive feelings do not always have to be absent for stress to grow, and vice versa for increases in capabilities (Garland et al., 2010). People may feel both positive and negative emotions at once in daily life because there are two separate cycle systems: upward spirals induced by favorable feelings and downward spirals resulting from negative feelings (Trampe et al., 2015).

Resources may be developed to help teachers deal with job obstacles or increase their favorable opinion of their teaching work whenever they are feeling good in the classroom. Conversely, when an instructor is having unpleasant feelings in a classroom environment, this leads to a continuous cycle of unpleasant feelings and causes lower levels of involvement in the classroom (Miller et al., 2008). According to Fredrickson (2013), the possible “undo effect,” which suggests that pleasant emotions may aid in stress recovery, is one of the functions of feelings of joy. As a result, both positive and negative aspects—such as psychosomatic stress, depression, and emotional exhaustion—are included in the concept of well-being. Examples of good aspects include work satisfaction, professional dedication, and engagement (Huppert and So, 2013; Collie and Martin, 2017). According to JD-R theory, feeling emotionally exhausted results in less participation and effort put into lesson planning as well as more unfavorable views toward learners (Frenzel et al., 2021). Emotional weariness can also negatively affect students’ academic performance as a result of increasing absence rates and instructor withdrawal from the classroom (Miller et al., 2008). On the other hand, instructors who are more emotionally involved tend to put more effort into the instruction and show more empathy and concern for their pupils. Committed instructors devote effort to investigating and improving their methods, and they may be better at handling classroom dynamics and enhancing their instruction quality, which benefits student results (Frenzel et al., 2021; Granziera et al., 2023).

### Growth mindset

Dweck (2006) established the concept of mindsets, or implicit/lay theories. According to Mercer and Ryan (2010), mindsets are one’s basic assumptions about various human characteristics, such as personality traits. It is commonly acknowledged that teachers’ perspectives have a significant impact on their professional success (Dweck, 2014). Additionally, these perspectives have a tendency to shape and reshape teachers’ perceptions of both success and failure in terms of how they approach overcoming various problems in L2 teaching (Cacali, 2019). The notion of mindset is highly significant in L2 education because the learning process itself is complicated (Nguyen et al., 2022). Fixed and growth mindsets are the two kinds into which mindsets fall (Dweck et al., 1995). A growing mindset primarily deals with the idea that the previously mentioned components are changeable and can be improved, whereas a fixed mindset is associated with the view that one’s talents, qualities, character, and IQ are fixed and cannot be modified (Haimovitz and Dweck, 2017; Dweck and Yeager, 2019).

Growth mindsets, which are consistently linked to actions, views, aspirations, and learning techniques, relate to a person’s shifting ideas about his or her own talents while dealing with failures (Molden and Dweck, 2006; Dweck, 2015; Schroder et al., 2019). Language mindset is concerned with individual viewpoints towards the malleability of language acquisition (Shirvan et al., 2021; Zhang et al., 2022). Investigators studying L2 who are interested in determining whether people view their characteristics as fixed or malleable have lately focused a lot of emphasis on studies on people’s opinions about their ability to acquire a second or foreign language (Lou and Noels, 2019; Liu et al., 2023). The inspiring factors of autonomy, well-being, perseverance, willingness to communicate, and speaking anxiety are only a few examples of the various emotional aspects that shape mindsets (Hochanadel and Finamore, 2015; Henriksen et al., 2018; Lou and Noels, 2019; Zeng et al., 2019; Zarrinabadi et al., 2021; Ozdemir and Papi, 2022).

Furthermore, according to Yeager et al. (2022), it is contended that a person’s feelings can change in relation to a variety of topics that are divided into growth and fixed mindsets. A growth mindset is used to describe instructors who are flexible, while a fixed mindset is used to describe those who are not. Also, personal characteristics and individual teaching styles are seen by instructors with a growth mindset as dynamic phenomena (Heggart, 2015). Contrarily, as asserted by Zeng et al. (2019), those with fixed mindsets view personality traits and talents as unchangeable, but people with growth mindsets consider that intelligence and ability can be developed. Zhang et al. (2017) summarized the studies on the connection between students’ academic accomplishment, their mindsets, and instructors’ mindsets that were published between 1998 and 2017 in order to determine the importance of this concept in educational settings. It was shown that instructors’ mindsets only played a mediating, not a deciding, role in the academic accomplishment of learners, notwithstanding the fact that students’ mindsets clearly played an essential role in this.

Moreover, Zeng et al. (2019) asserted that work engagement may be predicted by growth mindset, wellbeing, and effort persistence. According to Yeager and Dweck’s (2020) findings, it may be claimed that growth mindset’s benefits are reproducible, productive, and conceptually well-documented for both instructors and students. In another study, Derakhshan et al. (2022) examined the associations between classroom atmosphere, growth mindset, and the involvement of learners in a different study where boredom was used as a mediator. The results showed that students’ growth mindsets had an indirect influence on their involvement, and boredom with the classroom environment was the most important determinant of engagement. In a different study by Patrick and Joshi (2019), it was proposed that instructors’ preexisting beliefs about students and learning had an effect on their mentality. It was shown that students’ attitudes toward their own abilities, personalities, and characteristics are highly associated with teachers’ growth mindsets. The researchers also discovered that instructors’ perspectives might improve or worsen students’ perceptions of their level of ability or intellect.

Additionally, according to Frondozo et al. (2020), a teacher’s perspective and emotions might influence how engaged they are at work. Also, the results demonstrated that instructors who had a growth teaching mentality had better levels of work engagement, with enjoyment of the profession as a mediating factor. Besides, Irie et al. (2018) examined instructors’ perspectives on their own line of work. They discovered that while interpersonal abilities were seen as being natural, instructors were more concerned with their own technical expertise. Finally, research on the attitudes of future teachers at the start of their practicum was carried out by Gutshall (2014). The results showed that they maintained a consistent mentality throughout their training.

### Grit

According to Duckworth et al. (2007), “grit involves exerting diligent effort in the face of challenges, persisting and maintaining interest even in the presence of setbacks, difficulties, and periods of stagnant progress” (pp. 1087–1,088). Two characteristics—perseverance in work and constancy in interest—help define grit. The first one relates to what appears to be a propensity for investing consistent energy over an extended period of time. The second one is the consistency of a passion for larger-scale objectives despite becoming fixated while overcoming setbacks and difficulties (Sudina et al., 2021; Teimouri et al., 2022). According to Duckworth et al. (2007), gritty people are dedicated to their careers and rarely change jobs. Sudina et al. (2021) define “teachers’ grit” as their persistent enthusiasm for their work despite feeling tired, while overcoming inherent obstacles in the profession. According to research by Duckworth et al. (2009), grit and life satisfaction were important predictors of instructors’ ability to deliver successful lessons. Gritty instructors are more likely to be good teachers because they see teaching as a fun pastime (Maiers and Sandvold, 2017). Additionally, based on the study by Zeng et al. (2019), instructors who exhibit greater levels of grit have a growth mindset that aids them in overcoming obstacles.

In the domain of applied linguistics, limited research has been done on language instructors’ grit, especially in EFL contexts, despite the fact that L2 learners’ grit has received a lot of attention (Kazemkhah Hasankiadeh and Azari Noughabi, 2022; Teimouri et al., 2022). Sudina et al. (2021) developed the idea of language instructor L2 grit as a personality characteristic that impacts the degree to which teachers keep their desire to achieve the best of their abilities in response to the demand for domain-specific grit research. To measure the L2 grit of language instructors, the researchers developed a domain-specific instrument called the L2-Teacher Grit Scale. They contended that instructor L2 grit entails constancy of interest and persistence of effort.

Finally, it has been suggested that L2 grit has a beneficial association with foreign language fulfillment, dedication, growth mindset, a desire to interact, inspiration, and a negative relationship with stress and a fixed mindset (Teimouri et al., 2022). The development of greater psychological training for language instructors may be facilitated by studies on instructor L2 grit (Mercer, 2021; Derakhshan et al., 2022). Azari Noughabi et al. (2022) found a strong correlation between L2 grit, immunity, and job engagement in EFL instructors. In light of the fact that instructors’ enthusiasm and tenacity might result from how much they like their jobs, L2 grit may be suggested to be directly related to foreign language teaching enjoyment (FLTE) among language instructors (Lu, 2019). Besides, Zhao et al. (2018) concluded that the association between grit and growth mindset is somewhat mediated by learning incentives.

### Mindfulness

Researchers have defined mindfulness as a conscious awareness of an immediate, composable event (Li, 2021). Kabat-Zinn (2003), an expert in the study of mindfulness, describes mindfulness as “a state or quality of mind that arises through paying attention on purpose, in the present moment, and non-judgmentally to the unfolding of experience moment by moment” (p. 145). Since it concentrates on continuing living events, mindfulness may be seen as a dynamic procedure rather than a static outcome of action (Song and He, 2021). According to Brown and Ryan (2003), mindfulness is the understanding of current incidents and sensations that may be taken in with attention. As a unique and complicated construct, mindfulness may be thought of as a notion comprised of several components. It has both personal and professional components that enable instructors to relate to many aspects of life experiences (Song and He, 2021; Yuan et al., 2023). Time, evolution, methods of instruction and learning, professional growth, and the setting of L2 instruction are some of the crucial characteristics that Yuan et al. (2023) discovered to be related to the idea of mindfulness.

According to Sharp and Jennings (2016), instructors’ mindfulness promotes the creation of beneficial connections with learners, has an effect on their psychological variables, and advances their professional growth, all of which may result in pupils achieving more. It’s possible to learn and teach mindfulness, and instruction in mindfulness involves a range of activities, from regular practices to lengthy training programs (Song and He, 2021). It has been demonstrated that mindfulness training has a positive impact on emotional and psychological variables such as wellbeing, tension, and focus, and the practice of mindfulness has been demonstrated in clinical and health settings as well (Brown and Ryan, 2003; Lutz et al., 2008; Li, 2021). As mentioned by Jennings and Greenberg (2009), mindfulness training makes instructors more resilient and upbeat in difficult situations, which benefits students’ academic performance. Additionally, based on the study by Schussler et al. (2016), mindfulness training improves teachers’ effectiveness on the job. According to Altan et al. (2019), for both students and instructors, mindfulness training can result in benefits that are both immediate and permanent.

Moreover, Chiesa et al. (2011) examined the psychological well-being and mindfulness of instructors. Based on the findings, mindfulness training boosted psychological capabilities and coping mechanisms, which in turn enhanced resilience. Instructors’ prolonged stress and burnout may result in student-teacher interactions that have a negative impact on students’ performance. It is also a practical construct that boosts trainees’ efficiency and work satisfaction (Kidger et al., 2016). Also, Hwang et al. (2017) concluded that mindfulness training has a positive impact on teachers’ instructional effectiveness and general well-being. Finally, Xue (2023) mentioned that being mindful may be a powerful way to improve focus and awareness in the classroom. It also enables teachers to manage their time more effectively, which helps students become more aware of the present moment.

## The structural model

**Figure 1:**
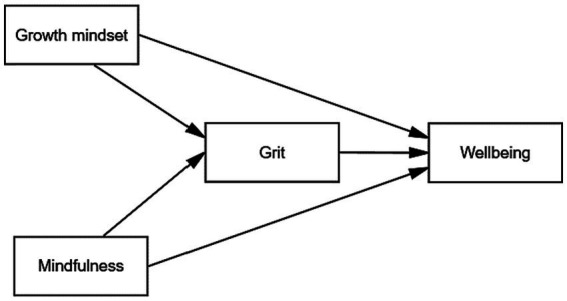
The structural model.

Given the theoretical and empirical background, a number of hypotheses were formulated for the structural model (see Figure 1):

*Hypothesis 1*: Teacher growth mindset is positively and directly related to teacher grit.

Research by Dweck (2006) has shown that individuals with a growth mindset believe that abilities can be developed through effort and learning. This mindset fosters a belief in the potential for improvement and a willingness to persist in the face of challenges (Zhao et al., 2018). Grit, on the other hand, aligns with the mindset of continuously striving for improvement (Dweck and Yeager, 2019). Studies by Duckworth et al. (2007) and Yeager and Dweck (2020) have demonstrated that individuals with a growth mindset tend to exhibit higher levels of grit. Therefore, based on this evidence, it might be warranted to propose that teacher growth mindset is positively related with teacher grit as reported by some studies in the literature (e.g., Keesey et al., 2018; Zeng et al., 2019).

*Hypothesis 2*: Teacher mindfulness is positively and directly associated with teacher grit.

Mindfulness involves being fully present and engaged in the current moment, cultivating awareness and non-judgmental acceptance (Bishop et al., 2004; Emerson et al., 2017). This state of mind can enhance self-regulation, emotional resilience, and perseverance among teachers (Roeser et al., 2012; Crain et al., 2017; Hwang et al., 2017), which are key components of grit. As such, it is hypothesized that there might be a positive association between mindfulness and grit, suggesting that individuals who are more mindful may also demonstrate higher levels of grit (Vela et al., 2018).

*Hypothesis 3*: Teacher grit is positively and directly related with teacher occupational well-being.

Grit involves a combination of passion and perseverance toward long-term goals, promoting a sense of purpose and fulfillment (Duckworth et al., 2009). Research has demonstrated that individuals with higher levels of grit experience greater well-being, including higher life satisfaction and lower levels of stress and burnout (e.g., Lan and Moscardino, 2019; Lan and Zhang, 2019; Yang, 2021). Given this background, it is justifiable to propose that teacher grit is positively associated with teacher well-being.

*Hypothesis 4*: Teacher grit mediates the relationship between teacher growth mindset and teacher occupational well-being.

Numerous studies have demonstrated the positive impact of a growth mindset on teacher well-being (e.g., Ortiz Alvarado et al., 2019; Nalipay et al., 2022; Liu et al., 2023). A growth mindset refers to the belief that abilities and intelligence can be developed through dedication and effort. Teachers with a growth mindset are more likely to perceive challenges as opportunities for growth, embrace feedback, and persist in the face of setbacks. This mindset fosters resilience, motivation, and a sense of self-efficacy, which are all crucial components of teacher occupational well-being (Han and Stieha, 2020; Shoshani, 2021). Teachers who possess a growth mindset tend to experience higher levels of job satisfaction, engagement, and overall well-being. Likewise, grit, characterized by perseverance and passion for long-term goals, has been consistently linked to positive outcomes, including well-being (Shao, 2023). Gritty individuals are more likely to maintain effort and overcome obstacles, even when faced with challenges or setbacks (Duckworth et al., 2007). In teaching context, grit enables teachers to persist in their profession, invest in their students’ learning, and navigate the demands of the job effectively. Teachers with high levels of grit tend to experience greater job satisfaction, professional fulfillment, and overall occupational well-being (Lan and Moscardino, 2019; Liu et al., 2023).

*Hypothesis 5*: Teacher grit mediates the relationship between teacher mindfulness and teacher occupational well-being.

Research has shown that mindfulness practices have a positive impact on teacher occupational well-being by reducing stress, improving emotional regulation, and enhancing overall mental health (Shapiro et al., 2006; Roeser et al., 2012; Hwang et al., 2017). Teachers who cultivate mindfulness may experience lower levels of burnout, greater job satisfaction, and improved overall well-being (Crain et al., 2017). In addition, gritty individuals demonstrate a steadfast commitment to their goals and are willing to exert sustained effort despite challenges and setbacks (Duckworth et al., 2007). In teaching settings, teachers with high levels of grit are more likely to persist in their profession, overcome obstacles, and maintain a positive outlook even in the face of difficulties (Lan and Zhang, 2019; Kazemkhah Hasankiadeh and Azari Noughabi, 2022; Liu et al., 2023). These qualities are closely associated with enhanced occupational well-being, job satisfaction, and a sense of purpose.

## Method

### Participants

A total of 547 EFL teachers were enlisted from 12 middle schools and high schools in diverse provinces throughout China to take part in this research and complete the survey. The selected schools encompassed both urban and rural areas, ensuring a varied and inclusive sample. The age spectrum of the participants ranged from 21 to 54 years, with an average age of 27.32 (SD = 6.81). Among the participants, 407 (74.40%) were women, and 140 (25.59%) were men. Concerning their educational background, 367 (67.3%) EFL teachers held a postgraduate degree or higher, while the remaining 180 (32.7%) possessed a bachelor’s degree. The allocation of teachers across schools was as follows: four schools were situated in rural regions, while the remaining eight schools were located in urban settings. On average, each school had 45 participating teachers, with the number of teachers ranging from 30 to 60. The participants in this study represented a broad spectrum of teaching experience. The majority of teachers had accumulated over 10 years of teaching experience (n = 377, 69.1%), while 130 (23.6%) had 5 to 10 years of experience, and 40 (7.3%) had less than 5 years of experience.

### Instruments

#### The L2-teacher grit scale (L2TGS)

The participants’ L2 grit was assessed using the L2-Teacher Grit Scale (L2TGS), a measurement tool developed and validated by Sudina et al. (2021). This scale comprises 9 items that participants rated on a 5-point response scale, ranging from 1 (not like me at all) to 5 (very much like me). The scale consists of two components: consistency of interest (α = 0.76; six items) and perseverance of effort (α = 0.78; eight items). Sudina et al. (2021) verified the reliability and validity of the L2TGS as a suitable measure of grit for EFL teachers. A sample item is “I finish whatever I begin related to my teaching plans and goals.”

#### Occupational well-being scale

The well-being of teachers was measured using Warr’s (1990) Occupational Well-Being Scale (OWS), consisting of 12 items. This scale aims to evaluate teachers’ well-being across two dimensions: ill-being and well-being. Participants were asked to indicate the extent to which they experienced job-related feelings of anxiety (feeling tense, uneasy, and worried), contentment (feeling calm, contented, and relaxed), depression (feeling depressed, gloomy, and miserable), and enthusiasm (feeling cheerful, enthusiastic, and optimistic) in the past few weeks. The response options ranged from 1 (never) to 6 (all of the time). A sample item of the scale is “I can deal with just about any problem in my job.”

#### Mindfulness scale

To evaluate participants’ mindfulness, the *Mindfulness Attention Awareness Scale* (MAAS), developed by Brown and Ryan (2003), was utilized. This self-report measure gauges individuals’ inclination to be present and attentive to current experiences. The scale comprises 15 items, rated on a 6-point Likert scale ranging from ‘almost always’ (1) to ‘almost never’ (6). An example item is: “It is challenging for me to maintain focus on the present moment.” Previous studies (Fallah, 2017) have provided evidence supporting the reliability and validity of this scale.

#### Growth mindset scale

In this study, participants’ growth mindset was assessed using a Chinese adaptation of the 4-item Growth Mindset Inventory, initially developed by Dweck (2006). The Chinese version of the inventory demonstrated favorable reliability and validity (Zeng et al., 2016). Responders rated the items on a 5-point Likert scale, ranging from 1 = (strongly disagree) to 5 = (strongly agree). For instance, an item stated, “*You can always substantially change how intelligent you are*.”

### Procedures

To ensure a comprehensive and ethically sound data collection process, this study followed several stages. Prior to commencing the study, ethical approval was obtained from the appropriate institutional review board. This approval confirmed that the study adhered to ethical guidelines and safeguarded the rights and confidentiality of the participants.

A total of 12 secondary schools from different provinces across China were purposefully selected for participation in this study. The selection aimed to encompass a diverse range of educational settings, including both urban and rural schools. Invitations were extended to 547 teachers from the selected schools, and their cooperation was sought with the assistance of school administrators. An information session was conducted to provide an overview of the study’s objectives and procedures, emphasizing that participation was voluntary. During this session, teachers were given the opportunity to seek clarification and address any concerns they may have had.

Informed consent forms were distributed to interested teachers prior to data collection. These forms provided detailed information about the study’s purpose, procedures, and potential risks and benefits. Participants were given ample time to review the forms and seek further clarification before providing their informed consent. This ensured that their participation was voluntary and based on a clear understanding of the study.

The data collection process involved the administration of a structured questionnaire comprising validated scales and measures to assess teacher well-being, growth mindset, grit, and mindfulness. Participants were allocated sufficient time to complete the questionnaire, and it was administered during their regular working hours. Throughout the data collection period, the researchers were available to address any inquiries or provide further explanations as needed. Completed questionnaires were collected and securely stored, with each questionnaire assigned a unique identification code to ensure anonymity and maintain confidentiality. Subsequently, the data were entered into a secure electronic database for analysis.

### Data analysis

The analysis of the data commenced with descriptive and correlational examinations. To test the research hypothesis, Structural Equation Modeling (SEM) was conducted using Amos version 26, while the SPSS (Ver. 25) was utilized for the other analyses. Following the recommendation of Anderson and Gerbing (1988), the measurement model was first fitted to the data, after which the underlying structural model was examined. In evaluating the overall fitness of the hypothesized model, multiple fit indices were considered. These included: (a) the ratio of *χ*^2^ to the degrees of freedom, (b) the Goodness of Fit Index (GFI), (c) the Comparative Fit Index (CFI), (d) the Root-Mean-Square Error of Approximation (RMSEA), and (e) the Standardized Root-Mean-Square Residual (SRMR). According to Kline (2023), a good fit is indicated by a small *χ*^2^ to the degrees of freedom value with *p* > 0.05. Additionally, GFI and CFI values equal to or higher than 0.90 (Hu and Bentler, 1999) suggest a good fit, while RMSEA values should not exceed 0.08, and SRMR values should not surpass 0.10 (Bowen and Guo, 2011).

## Results

The descriptive analysis of the study variables is presented in Table 1. The participants reported a mean growth mindset score of 3.48 (SD = 0.71) and a Cronbach’s α coefficient of 0.784. Mindfulness had a mean score of 3.86 (SD = 0.68) with a Cronbach’s α of 0.806. Grit had a mean score of 3.12 (SD = 0.87) and a Cronbach’s α of 0.886. Wellbeing had the highest mean score of 4.07 (SD = 0.79) with a Cronbach’s α of 0.824.

**Table 1:** Descriptive analysis.

	Mean	SD	Croanbach’s α	1	2	3	4
1. Growth mindset	3.48	0.71	0.784	1			
2. Mindfulness	3.86	0.68	0.806	0.363^**^	1		
3. Grit	3.12	0.87	0.886	0.674^**^	0.426^**^	1	
4. Wellbeing	4.07	0.79	0.824	0.476^**^	0.293^**^	0.517^**^	1

^**^p-value <0.01.

The correlation matrix (Table 1) presents the pairwise correlations between the variables. Growth mindset and mindfulness exhibited a significant positive correlation (*r* = 0.363, *p* < 0.01), indicating that individuals with stronger growth mindset beliefs tended to have higher levels of mindfulness. Grit and growth mindset were highly positively correlated (*r* = 0.674, *p* < 0.01), suggesting that those with greater grit also tended to hold stronger growth mindset beliefs. Additionally, grit and mindfulness displayed a positive correlation (*r* = 0.426, *p* < 0.01), indicating a connection between perseverance and mindfulness. Well-being showed positive correlations with growth mindset (*r* = 0.476, *p* < 0.01), mindfulness (*r* = 0.293, *p* < 0.01), and grit (*r* = 0.517, *p* < 0.01), underscoring the importance of these factors in contributing to overall well-being.

To assess the validity of the measurement models, confirmatory factor analysis (CFA) was conducted. The factor loadings of all items in the CFA model are provided in Table 2. Adjustments were made as certain measurement models did not meet the recommended level of data adequacy. Upon examination of the results, it became apparent that one item related to grit (Gr6), one item related to wellbeing (WB9), and two items related to mindfulness (M2 and M9) exhibited factor loadings below the established threshold of 0.5. Following the suggestion by Hair et al. (2010), these items were excluded from the model. Subsequently, the final model was derived after incorporating these modifications to the original design. The results of the final CFA model indicated an acceptable fit, with χ2/df = 2.46, GFI = 0.92, CFI = 0.94, RMSEA = 0.06 (90% CI: 0.05–0.07), TLI = 0.92, and SRMR = 0.07, providing support for the construct validity of the measurement models.

**Table 2:** The results of measurement model testing.

Variable	Indicators	AVE	MSV	ASV	Cronbach’s a/CR	Factor loadings
Grit	Gr1	0.59	0.52	0.42	0.872/0.872	0.69^***^
	Gr2					0.72^***^
	Gr3					0.81^***^
	Gr4					0.56^***^
	Gr5					0.67^***^
	Gr7					0.80^***^
	Gr8					0.85^***^
	Gr9					0.66^***^
	Gr10					0.53^***^
	Gr11					0.72^***^
	Gr12					0.76^***^
	Gr13					0.71^***^
	Gr14					0.69^***^
Wellbeing	WB1	0.64	0.59.	0.47	0.792/0.792	0.63^***^
	WB2					0.81^***^
	WB3					0.92^***^
	WB4					0.86^***^
	WB5					0.85^***^
	WB6					0.77^***^
	WB7					0.58***
	WB8					0.66***
	WB10					0.61***
	WB11					0.73^***^
	WB12					0.76^***^
Mindfulness	M1	0.54	0.52	0.38	0.814/0.814	0.81^***^
	M3					0.73^***^
	M4					0.76^***^
	M5					0.80^***^
	M6					0.77^***^
	M7					0.54^***^
	M8					0.58^***^
	M10					0.62^***^
	M11					0.74^***^
	M12					0.80^***^
	M13					0.73^***^
	M14					0.83^***^
	M15					0.90^***^
Growth mindset	GM1	0.55	0.53	0.41	0.761/0.761	0.72^***^
	GM2					0.84^***^
	GM3					0.78^***^
	GM4					0.62^***^

AVE indicates average variance extracted; MSV shows maximum shared variance; ASV shows average shared variance; CR indicates construct or composite reliability. ^***^ significant at the 0.001.

Table 2 demonstrates that all the measures’ coefficients exceeded 0.70, thus confirming their sufficient reliability. Composite or construct reliabilities ranged from 0.761 (growth mindset) to 0.872 (grit). The factor loadings of all scales demonstrated statistical significance (*p* < 0.001) and fell within acceptable ranges. These findings indicated strong construct reliabilities and significant factor loadings, which offer empirical support for the model’s convergent validity (Anderson and Gerbing, 1988). Additionally, the average variance extracted (AVE) values were consistently above 0.5, and composite reliabilities exceeded the AVE values. Therefore, these findings further confirmed the convergent validity of the model (Hair et al., 2010).

Discriminant validity was also examined in this study. According to Fornell and Larcker (1981), the AVE value of each construct should surpass the squared correlation coefficient with other constructs. Table 3 provided evidence of discriminant validity. Furthermore, by considering the maximum shared variance (MSV) and average shared variance (ASV) in conjunction with the AVE values, it was observed that all the ASV and MSV values were lower than their respective AVE values, supporting the presence of discriminant validity (Hair et al., 2010). Additionally, in order to examine the issue of common method bias, the researchers conducted Harman’s one-factor test (Podsakoff and Organ, 1986). The results of this test indicated that the first factor accounted for 43.58% of the variance, which was below the 50% threshold. As a result, it can be concluded that common method bias was not a major concern in this research (see Table 4).

**Table 3:** Discriminant validity.

	1	2	3	4
1. Growth mindset	**0.55**			
2. Mindfulness	0.13	**0.54**		
3. Grit	0.45	0.18	**0.59**	
4. Wellbeing	0.22	0.08	0.26	**0.64**

Bold-faced values are square roots of the AVE; off diagonals are correlation coefficients.

**Table 4:** Results of fit indices of structural models.

Model	χ^2^	df	Δχ^2^	GFI	CFI	RMSEA	TLI	SRMR
Direct Effect Model (C)	1413.762^**^	746	–	0.821	0.912	0.066	0.924	0.166
Full Mediation Model (B)	1294.514^**^	742	119.248	0.879	0.947	0.046	0.934	0.091
Partial Mediation Model (A)	1098.479^**^	735	196.035	0.899	0.971	0.034	0.966	0.071

Δ*χ*^2^ shows differences between model and the subsequent model. ^**^p-value < 0.001.

### Top of form

After confirming the satisfactory fit of the measurement model, several competing structural models were examined to test the study hypotheses. Initially, the hypothesized partial mediation model (Model A) was compared with the full mediation model (Model B), where all path coefficients from teacher growth mindset and mindfulness to wellbeing were set to zero. Additionally, a competing direct model (Model C) was tested, constraining all path coefficients to and from teacher grit to zero. The goodness-of-fit statistics for all three alternative structural models are presented in Table 5. Overall, the fit indices of the hypothesized model demonstrated significantly better fit than both Model B (df = 7, Δ*χ*^2^ = 196.035, *p* < 0.001) and Model C (df = 11, Δ*χ*^2^ = 315.283, *p* < 0.001). The fit indices were all found to be significant. Consequently, Model A (partially mediated model) was retained as the best fitting model.

**Table 5:** Path estimates of structural model.

Standardized path coefficients (*t*-value)			
	Direct effects model	Full mediation model	Partial mediation model
Growth mindset → wellbeing	0.316 (4.56^**^)		0.287 (3.93^**^)
Mindfulness → wellbeing	0.173 (2.36^*^)		0.112(1.37^*^)
Growth mindset → grit		0.496 (7.08^***^)	0.428 (6.92^***^)
Mindfulness → grit		0.372 (4.66^**^)	0.335 (4.39^**^)
Grit → wellbeing		0.451 (7.14^***^)	0.436 (6.57^***^)

^*^p-value <0.05, ^**^p-value <0.01, ^***^p-value <0.001.

**Figure 2:**
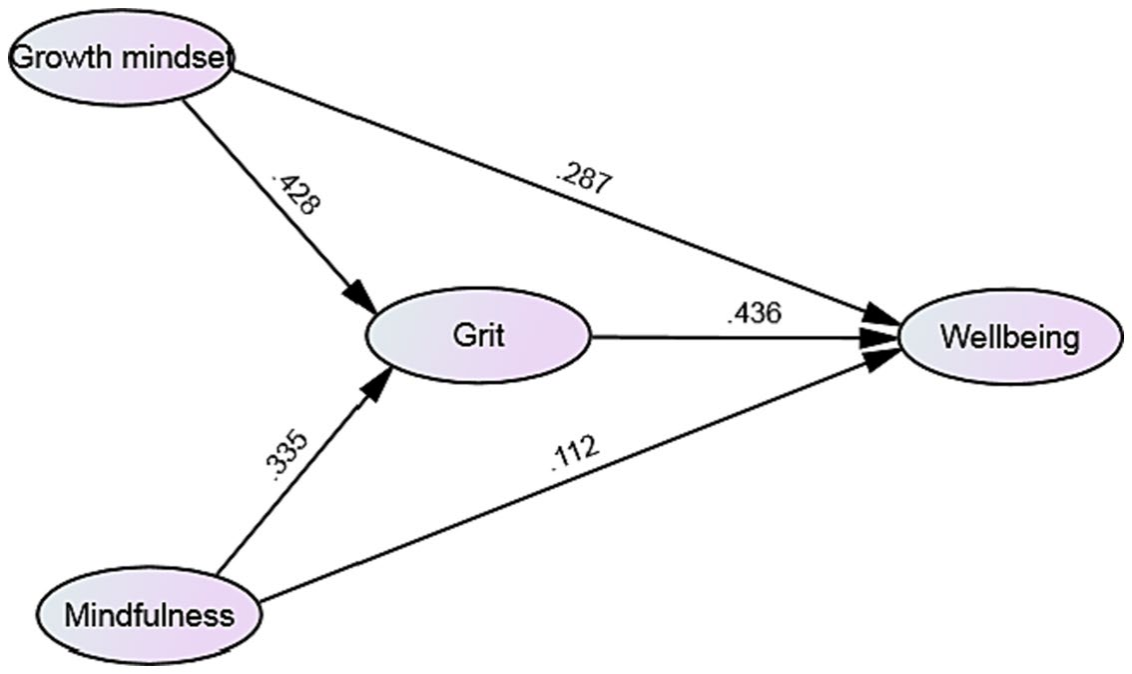
The Structural model.

Figure 2 illustrates the path and parameter estimates for the final partially mediated model (Model A). All path coefficients were found to be statistically significant, although the impact of mindfulness on wellbeing was observed to be small (*β* = 0.112, *p* < 0.05). The structural model provided empirical support for H1, as growth mindset significantly influenced teacher grit (*β* = 0.428, *p* < 0.001). Similarly, mindfulness exhibited a significant positive association with grit (*β* = 0.335, *p* < 0.001), thereby confirming H3. Furthermore, teacher grit showed a positive relationship with wellbeing (*β* = 0.436, *p* < 0.001), thus supporting H3.

Finally, we employed Baron and Kenny’s (1986) method to investigate whether teacher grit acted as a mediating variable among the study variables. In the direct model (see Table 5), significant path coefficients were observed between teacher growth mindset and mindfulness on wellbeing (growth mindset → wellbeing: 0.287, *p* < 0.01; mindfulness → wellbeing: 0.132, *p* < 0.05), thus confirming the initial step of Baron and Kenny’s test. Moving on to the full mediation model, significant path coefficients were found between growth mindset and mindfulness on grit (growth mindset → grit: 0.428, *p* < 0.001; mindfulness → grit: 0.335, *p* < 0.01), validating the second step of Baron and Kenny’s method. Lastly, in the partial mediation model, growth mindset exhibited a positive influence on wellbeing (*β* = 0.287, *p* < 0.01). In conjunction with the indirect effect of grit, growth mindset had a positive impact of 0.186 (0.428 × 0.436) on wellbeing, which was lower than the direct influence (0.186 < 0.287) of growth mindset on wellbeing. Consequently, grit partially mediated the relationship between growth mindset and wellbeing, partially supporting H4. Additionally, mindfulness displayed a small yet significant path coefficient on wellbeing (*β* = 0.112, *p* < 0.05). Teacher grit was identified as a complete mediator between mindfulness and wellbeing (0.335 × 0.436 = 0.146 > 0.112). Thus, the influence of mindfulness on grit enhanced wellbeing, providing full support for H5.

## Discussion

The current study aimed at exploring the relationship between teacher growth mindset, mindfulness, grit, and teacher well-being, with a particular focus on the mediating role of grit in the Chinese EFL context. It has several findings that will be discussed below.

Firstly, it was concluded that teacher growth mindset is positively and directly related with teacher grit. This finding is in accordance with previous research that highlight the important role of mindset in boosting grit (e.g., Hochanadel and Finamore, 2015; Keesey et al., 2018; Zhao et al., 2018). In line with Zhao et al. (2018), the present study explained that individuals with a growth mindset are more likely to develop learning motives that are more independent and self-directed, which increases their level of grit. These results underline how crucial it is to instill a growth mindset among teachers. Instructors who believe that their cognitive skills, competence, and other qualities can be enhanced through self-effort might be less susceptible to being under the control of others and might develop a stronger sense of autonomy through self-motivation driven by standards, purpose, enthusiasm, and identity as individuals. They are more likely to have a higher propensity to continue in the face of difficulties, and a lower propensity to give up on something they are passionate about in the face of numerous distractions. Additionally, nurturing such an attitude might have long-term advantages that go beyond improved academic instruction. Moreover, it was found that a teacher’s growth mindset affects their well-being. This finding showed that adopting a growth mindset might contribute to greater levels of well-being, which subsequently had a favorable impact on instructors’ work engagement (Zeng et al., 2019).

Second, it was found that teacher grit was positively and directly associated with teacher occupational well-being. This finding can be supported by Sudina et al. (2021), who demonstrated a strong link between instructors’ L2 grit and their well-being, meaning that hardworking FL instructors might be more psychologically motivated to achieve their professional goals and mentally resolved to overcome failures. They might be less likely to feel fatigue since they have such a positive mindset toward goal achievement. Gritty individuals tend to have a strong sense of purpose and meaning in their work, which is associated with higher levels of life satisfaction and well-being (Lan and Moscardino, 2019). They are more likely to experience a sense of accomplishment and fulfillment, as they persevere and make progress towards their goals (Lan and Zhang, 2019). Furthermore, grit is closely related to self-regulation and self-control, which are important factors in maintaining well-being (Kazemkhah Hasankiadeh and Azari Noughabi, 2022). Gritty individuals demonstrate the ability to delay gratification, manage their emotions, and engage in adaptive coping strategies (Duckworth and Gross, 2014). These skills contribute to their occupational well-being by reducing stress, promoting emotional stability, and enhancing their overall ability to navigate challenging situations (Yang, 2021; Liu et al., 2023; Shao, 2023).

Third, it was revealed that teacher grit mediated the relationship between teacher mindfulness and teacher occupational well-being. According to previous research (e.g., Brown and Ryan, 2003; Hwang et al., 2017), mindfulness can significantly influence well-being. Mindfulness practices have been associated with various positive outcomes, including reduced stress, enhanced emotional well-being, and improved psychological functioning (Emerson et al., 2017). Mindfulness promotes self-awareness, self-regulation, and the ability to respond to challenges and stressors in a more adaptive manner (Shapiro et al., 2006). By cultivating mindfulness, teachers develop the ability to manage stress, regulate emotions, and maintain a positive mindset, leading to greater occupational well-being. Mindfulness practices can enhance teachers’ self-care, reduce burnout, and improve their overall satisfaction with their professional roles (Crain et al., 2017). Furthermore, mindfulness practices often involve cultivating a non-judgmental attitude towards one’s experiences, which can promote self-compassion and self-acceptance. This self-compassion, in turn, may contribute to the development of grit by providing a supportive internal environment that nurtures perseverance and resilience (Raphiphatthana et al., 2018, 2019; Jarukasemthawee et al., 2021). Thus, mindfulness may indirectly foster grit by enhancing self-compassion and creating a positive mindset that encourages sustained effort and goal pursuit.

Overall, it was revealed that teachers with a growth mindset are more likely to perceive challenges as opportunities for growth, leading to higher levels of job satisfaction and overall occupational well-being. Also, teachers with high levels of grit tend to experience greater job satisfaction, professional fulfillment, and overall well-being. Finally, when teachers are mindful, they are more likely to develop grit, which in turn contributes to their occupational well-being. These findings highlight the importance of cultivating growth mindsets, grit, and mindfulness among teachers to enhance their well-being and overall professional satisfaction.

Although our findings affirm existing knowledge, they also bring new dimensions to the discourse on teacher occupational well-being, growth mindset, mindfulness, and grit in EFL contexts. Notably, our study is one of the first efforts to comprehensively examine these constructs in a single model. Prior research has often explored these factors in isolation, overlooking the intricate inter-relations among them. In addition, one of the noteworthy novelties of our study is its focus on teacher mindfulness, a construct that has remained under-explored in the field of education, particularly within the realm of EFL teaching. Mindfulness, with its potential to influence professional well-being, resilience, and emotional regulation, emerges as a promising avenue for research in the educational context. By incorporating mindfulness into our model, we contribute to filling this research gap and offer a nuanced perspective on its interplay with growth mindset, grit, and teacher occupational well-being.

Our study goes beyond traditional linear models by examining the inter-relationships among these constructs. For instance, we demonstrate how teacher grit mediates the relationship between mindfulness and well-being, a nuanced finding not extensively covered in prior literature. This holistic approach broadens our understanding of the complex dynamics at play within the teaching profession. Given the relative novelty of our approach and the unique insights it has unveiled, we encourage future research to further delve into the intricate connections between these constructs. Explorations in diverse educational and cultural contexts can provide a more comprehensive understanding of the universal and context-specific aspects of teacher occupational well-being, mindfulness, growth mindset, and grit.

## Conclusion

In conclusion, this study sheds light on the complex interplay between teacher characteristics and well-being in the context of education. The findings highlight the significance of teacher growth mindset, grit, and mindfulness in predicting and influencing teacher occupational well-being. The direct influence of growth mindset and grit on well-being emphasizes the importance of fostering beliefs in personal growth and cultivating perseverance and passion in the teaching profession. Additionally, the mediating role of grit in the relationship between mindfulness and well-being suggests that developing mindfulness practices can contribute to the cultivation of grit, which in turn positively impacts teacher well-being.

The findings of this study have significant implications for educational contexts, teacher training, and the development of teacher support programs. Firstly, the positive relationship between teacher growth mindset and teacher occupational well-being emphasizes the importance of fostering a growth mindset among teachers. Efforts should be made by educators and policymakers to promote the belief that abilities and intelligence can be developed through effort and effective strategies. Professional development programs can be designed to equip teachers with the necessary tools and resources to cultivate a growth mindset. Secondly, the role of teacher grit in predicting teacher well-being highlights the value of perseverance and resilience in the teaching profession. To help teachers overcome challenges and maintain well-being, school administrators and teacher training institutions should provide targeted training programs and support mechanisms that build resilience, problem-solving skills, and stress management strategies. Additionally, the finding that teacher grit mediates the relationship between teacher mindfulness and well-being suggests that cultivating mindfulness can indirectly contribute to teacher well-being by fostering grit. Incorporating mindfulness practices and training into professional development programs can help teachers develop self-awareness, emotional regulation, and stress reduction techniques, ultimately promoting their well-being. In practical terms, educational institutions and policymakers should integrate strategies that promote growth mindset, grit, and mindfulness into teacher support programs, such as ongoing professional development, mentoring programs, and supportive work environments. By prioritizing these factors, the well-being and job satisfaction of teachers can be enhanced, leading to improved educational outcomes for students.

In this study, while we have gained valuable insights into the relationship between teacher growth mindset, mindfulness, grit, and occupational well-being, it is important to acknowledge certain limitations. Firstly, the cross-sectional design restricts our ability to establish causal relationships between the variables. Longitudinal or experimental designs would offer stronger evidence of causality and enable the examination of how these constructs evolve over time. Secondly, the use of self-report measures introduces the possibility of common method biases and response biases. Despite our efforts to ensure confidentiality and anonymity, social desirability bias may have influenced participants’ responses. Future research could incorporate multiple data sources, such as observer ratings or objective measures, to enhance the validity of the findings. Additionally, the exclusive focus on Chinese EFL teachers may limit the generalizability of the results to other cultural and educational settings. To develop a more comprehensive understanding, it is important to consider cultural factors and contextual variations in teacher well-being in future studies. Furthermore, while the selected measures for growth mindset, mindfulness, grit, and well-being have been validated, alternative measures or additional constructs could provide a more comprehensive assessment of the variables under investigation. Another potential limitation is the presence of common method bias due to relying solely on self-report measures for all variables. Although steps were taken to address this bias, it is essential to acknowledge the potential influence of shared method variance on the observed relationships. Moreover, the limited sample size of this study may impact the statistical power and generalizability of the findings. Future studies with larger and more diverse samples would enhance the robustness and external validity of the results. Lastly, it is important to recognize that other factors, such as organizational support, job demands, and personal characteristics, may also contribute to teacher well-being. Future research should explore the intricate interplay between these factors to provide a more comprehensive understanding of teacher well-being.

## Data availability statement

The data analyzed in this study is subject to the following licenses/restrictions: The raw data supporting the conclusions of this article will be made available by the authors, without undue reservation. Requests to access these datasets should be directed to JH: hejianyu_ly@hotmail.com.

## Ethics statement

The studies involving humans were approved by School of Foreign Languages, Luoyang Normal University, Luoyang 471,000, Henan, China. The studies were conducted in accordance with the local legislation and institutional requirements. The participants provided their written informed consent to participate in this study.

## Author contributions

All listed authors have made equitable and substantial intellectual contributions across every facet of this work, including conceptualization, methodology development, literature review, drafting, revisions, data collection, participant recruitment, data analysis, editing, research oversight, and other essential contributions. Together, they have granted unanimous approval for its publication.
